# Lived experiences of women during induction of labour at a tertiary hospital in Ghana: A qualitative study

**DOI:** 10.1371/journal.pgph.0002290

**Published:** 2024-02-15

**Authors:** Kenneth Atobrah-Apraku, Grace T. Newman, Yaw Opuni-Frimpong, Joseph D. Seffah, Kwame Adu-Bonsaffoh

**Affiliations:** 1 Department of Obstetrics and Gynaecology, Korle-Bu Teaching Hospital, Accra, Ghana; 2 Department of Obstetrics and Gynaecology, University of Ghana Medical School, Accra, Ghana; PLOS: Public Library of Science, UNITED STATES

## Abstract

Induction of labour (IOL) has become a major and vital maternal health intervention to facilitate childbirth and minimize the rising caesarean section rates globally. However, there is limited information to facilitate appropriate client counselling, birth preparedness and informed decision making although the procedure has inherent tendency for adverse maternal/perinatal outcomes. Given the need for optimal client education and shared decision making in maternal health, this study explored women’s knowledge and their lived experiences of IOL. This qualitative study used in-depth interviews, conducted at the largest teaching hospital in Ghana. Purposive sampling was used to recruit the study participants. Data analysis was performed based on thematic content using inductive framework synthesis. We included 17 women who had undergone IOL, delivered and discharged. Most participants(52.9%) were ≥30 years old, married(88.2%), and 41.1% had no previous childbirth experience. The main indications of IOL were postdate(47%), pre-eclampsia(29%) and gestational diabetes mellitus(11.8%). Data synthesis resulted in three broad themes: women’s knowledge on IOL, women’s experiences of care and women’s difficult experiences including coping mechanisms. We determined mixed responses concerning the themes explored: adequate versus inadequate knowledge; positive versus negative experiences of care and satisfaction. Nearly all women mentioned vaginal examination as their most difficult experience due to severe pain, extreme discomfort, and being psychologically traumatic. The main coping strategy the women developed to navigate the traumatic vaginal examination was by “psyching” themselves. Our study indicates women encounter significant negative and positive experiences during IOL and childbirth in Ghana with vaginal examination cited as the most painful experience. Appropriate antenatal counselling, women empowerment and pre-labour education on childbirth processes and expectations are recommended to enhance birth preparedness and complication awareness. Health system improvement and regular refreshers courses for health workers are urgently required to promote positive women’s experiences of care during labour induction and childbirth.

## Introduction

Induction of labour (IOL) has been an integral part of obstetric practice since the late 1700s when it was performed for very limited indications. However, it has become a major and common maternal health intervention to facilitate childbirth and avert caesarean birth [1]. IOL, defined as the artificial initiation of labour before its natural onset, targets two main events that lead to childbirth–cervical ripening and uterine contractions [2]. Labour induction is usually undertaken when the risk of pregnancy prolongation till the natural labour onset outweighs the risk of immediate delivery. Recognized indications for IOL include prolonged pregnancy, premature rupture of membranes and medical conditions in pregnancy such as diabetes, pre-eclampsia and sickle cell disease [3–5]. However, IOL, in itself, is associated with significant maternal and perinatal risk. Common maternal complications of IOL include failure of the induction process, uterine hyperstimulation, caesarean section, uterine rupture and postpartum haemorrhage whiles perinatal risks comprise fetal distress, birth asphyxia, neonatal encephalopathy, neonatal infections like meningitis and sepsis [6].

Globally, IOL is widely practiced and there is adequate evidence that this obstetric intervention significantly reduces maternal-fetal morbidity and mortality [7]. In Africa, labour induction rates are much lower compared to high income countries [8, 9]. For instance, a recent World Health Organization (WHO) Global Survey reported the prevalence of IOL was about 1.4%, 6.3% and 6.8% of births in Niger, Nigeria and Angola respectively [10]. In Ghana, data on national incidence of IOL is limited; however, the WHO multi-country study on “how women are treated during childbirth in health facilities” reported the overall incidence in three facilities to be 15% [11]. The WHO Global Survey revealed that Africa has an unmet need for induction of labour of about 66–80%, with labour induction rate of approximately 4% [10]. However, various methods of IOL (pharmacological and non-pharmacological) are available with varying effectiveness and potential risks. The factors that influence the choice of method for IOL include cervical ripening and membrane status, parity, and patients’ and providers’ preferences [6]. Comparatively, a recent multi-country cross-sectional study indicates that IOL accounts for approximately 30% of births in high income countries with Ireland recording the highest rate of 35.9% [9].

Considering the risk-benefit balance, women’s acceptance of IOL can depend on their perspectives about the procedure including health workers who provide the care. There is evidence that women’s overall experience during childbirth shapes their perspectives, which are influenced by parity, pregnancy classes, labor pains, medical interventions, support received from partners and professionals, and a sense of being in control [12, 13]. Both positive and negative experiences have been reported in previous studies. Positive factors such as sense of control and involvement in decision-making are thought to boost women’s self-confidence and feelings of success, resulting in a smoother transition to motherhood and formation of strong links with their infants [13]. Negative experiences including emotional distress and dread of future pregnancies and childbirth have been linked to medicalized birth environment and unplanned obstetrical emergency interventions. This may result in postpartum depression and decrease in mothers’ ability to care for their infants [13].

In addition, it is not uncommon to find varying ranges of dissatisfaction in women who undergo induction of labour [14, 15]. Some have expressed dissatisfaction with IOL due to lack of adequate information from health providers. Others reported a significant disconnect between induction expectations and their actual experiences including experiences of significant constrain and long waiting times for the induction process [16], feeling of losing control [17], lack of adequate preparation and planning for the procedure and informed consent [15, 18, 19]. With the current discourse in health, patient-centered care is strongly encouraged especially in maternal and newborn health. Women need to be empowered adequately to make appropriate decisions based on informed choices and have adequate control of their reproductive health matters to facilitate positive pregnancy and childbirth experiences.

In Ghana, there is limited qualitative data on IOL, especially user experiences, challenges and quality of care. Previous quantitative studies have reported IOL success rates over 80% with vaginal misoprostol [20]. In another study, both sublingual and vaginal misoprostol were found to be effective in inducing women with intrauterine fetal death [21]. Given that labour induction can potentially be stressful to the mother, women’s lived experiences can contribute significantly to interventions and policies to improve birth outcome. For instance, frequent vaginal examination forms an integral part of IOL and there is evidence the women experience significant physical and psychological distress during vaginal assessment for labour progress [22–24]. Previous research on induction of labour has predominantly been quantitative, procedure focused and has not addressed the women’s perspectives and experiences of IOL. Against this backdrop, this study explored women’s perspectives and lived experiences of induction of labour in a tertiary hospital in Ghana.

## Methods

### Study design and site

This was a non-experimental, qualitative study conducted at the Maternity unit of the Korle Bu Teaching Hospital (KBTH) in Accra, Ghana, from September 2021 to October 2021. KBTH is Ghana’s largest hospital and a tertiary referral center conducting approximately 10,000 deliveries annually. Induction of labour accounts for significant proportions of deliveries in the hospital [5]. Generally, IOL is initiated by the medical doctors at the hospital and the midwives actively monitor the maternal and fetal conditions to ensure early detection and treatment of complications.

### Participant selection

The inclusion criteria consisted of mothers who had singleton gestations, aged ≥18 years who had experienced induction of labour and given birth at the KBTH. We excluded women who had been induced from other facilities before referral to KBTH and women who were below 18 years. Study participants were recruited via purposive sampling, a non-probability sampling characterized by identification and recruitment of appropriate research participants based on the objectives of the study [25]. One of the authors (GTN) identified the women who had been scheduled for IOL and the research assistant approached and recruited the participants via face-to-face interaction. Twenty participants were approached and three refused to participate in the study because they did not have time for the interview.

### Data collection

Following informed consent, we used in-depth interviews (IDIs) to obtain a thorough understanding of women’s experiences following induction of labour. The use of IDIs generates a comprehensive understanding of human phenomenon based on individual experiences and perspectives [26]. The interviews were conducted after the women had been discharged from the health facility using interview guide (S1 Text). The interviews were conducted by a research assistant with experience in conducting qualitative interviews. The research assistant was a female and had BSc degree. The participants and the interviewer had never met or had any prior relationship. The interviews were conducted in English or Twi (the major local language) and audio-recorded. Each interview lasted between 30 to 45 minutes on average. The interviews were conducted in private rooms on the maternity wards. The data collection and analysis continued until data saturation was reached when further interviews yielded no new themes.

### Data management and analysis

All audio interviews were translated and transcribed into English shortly after they ended, and the transcripts’ completeness and accuracy were double-checked and validated by the senior research fellow in the team. Data analysis was conducted based on thematic content. Coding was performed by two authors (KAA and KAB). In this study, inductive qualitative analytic framework approach was employed to identify common themes discussed by the respondents through repeated reading of the transcripts and generation of the relevant codes. In this study, wide-ranging women’s experiences were ensured by including women who had different indications for the labour induction such as postdate (prolonged pregnancy), pre-eclampsia and gestational diabetes. These women were also managed by different teams (five) of obstetricians at the Maternity unit of the hospital. We used the consolidated criteria for reporting qualitative research (COREQ) [27] as a guideline in article.

### Ethical consideration

We received ethical approval from the Ethical and Protocol Review Committee of the College of Health Sciences University of Ghana (Protocol ID: CHS-EtM.2-P5.5/2020-2021). In addition, we obtained written informed consent all participants prior to the in-depth interviews. Anonymity was ensured by non-inclusion of any identifiable data on the study participants.

## Results

The study included 17 women who had undergone induction of labour and given birth. Majority of the study participants (52.9%) were 30 years old or more, married (88.2%) and Christians (94.1%), and 41.1% had no previous childbirth experience. In addition, 35.3% of the women had completed tertiary education and 76.4% were of Akan ethnicity. In this study, induction of labour was undertaken for eight cases of postdate (47%), five pre-eclampsia cases (29%), two cases of gestational diabetes mellitus (11.8%) and one case of sickle cell disease (5.9%) and one case of fetal anomaly not compatible with life (5.9%). During labour, the women were provided with intermittent pethidine injections for pain relief (none of them had epidural analgesia). Five (29.4%) out of the 17 women had failed induction of labour and were delivered via caesarean section. The details of the socio-demographic characteristics are presented in Table 1.

**Table 1:** Socio-demographic characteristics of women who had induction of labour.

Variable	Number (n)	Percentage (%)
**Age (years)**		
<20	1	5.9
20–29	7	41.2
≥30	9	52.9
**Marital status**		
Single	2	11.8
Married/co-habiting	15	88.2
**Education**		
None/primary	2	11.8
Junior High School	6	35.3
Senior High School	3	17.6
Tertiary	6	35.3
**Number of previous births**		
0	7	41.2
1	4	23.6
2	3	17.6
≥3	3	17.6
**Religion**		
Christian	16	94.1
Muslim	1	5.9
**Ethnicity**		
Akan	13	76.4
Ewe	1	5.9
Ga	1	5.9
Other	2	11.8
**Occupation**		
Unemployed	1	5.9
Hairdresser	1	5.9
Trader	7	41.2
Seamstress	2	11.8
Others	6	35.2
**Previous induction of labour**		
Yes	1	5.9
No	16	94.1

The major themes that emerged from the thematic analysis include following:

1. Women’s knowledge on induction of labour
2. Women’s experiences of care
3. Women’s difficult experiences and coping strategies

### 1. Women’s knowledge on induction of labour

Women’s knowledge on induction of labour was an important theme that emerged from the interviews. There were mixed findings concerning women’s knowledge on labour induction with a significant proportion who had never heard of the procedure. Seven (41%) of the 17 respondents had no prior knowledge about induction of labour. The first time most of the women heard of IOL was the time the procedure was performed for them.

“*I hadn’t done one before*. *I knew nothing about it*. *Because for my first 2 children I delivered spontaneously*.”

*(31 years and married)*

“*I didn’t know anything about it*. *But they explained everything in details to me before it was done*.*”*

*(33 years and married)*“

*I did not know much*. *I had heard about it in conversations that it is painful*. *It was also explained to me before we started it though*.*”*

*(32 years and married)*

Nearly 60% (n = 10) of the participants had some knowledge about induction of labour prior to undergoing the procedure. They had either heard of the procedure from other people including health workers or read about it on the internet. Most of the respondents commented that they were adequately counselled prior to the commencement of the induction procedure. Some of the participants were aware of some of the medications used for IOL and the indications undergoing the procedure.

*“I heard about it from colleagues that it is called “forced labour” and I knew Cytotec is the drug they use*.*”*

*(36 years and married)*

*“I heard about it when my date passed so I read about it on the internet*.”

*(37 years and married)*

One woman who had previously delivered vaginally prior to the current childbirth indicated that she had limited information on induction of labour. Although she had heard about IOL, she only knew about the IOL provided via intravenous fluid but not intravaginal insertion of medications or through the oral route.

“*I delivered all my children myself without any intervention so I did not know how it was*. *I had heard of it before but I did not know what it was really about*. *I only thought it was about the drip that is given and not insertion of drugs under me or under my tongue*. *But they explained everything to me that they will insert in my “private part” to help my cervix open so that the baby can come out*.*”*

*(36 years and married)*

### 2. Women’s experiences of care

Women described varied experiences of care during induction of labour and childbirth. Few mothers indicated their displeasure with the care they received in the hospital including the labour induction process. For instance, one mother recounted her experience of suboptimal quality of care especially with the night nurses. She narrated that there was significant delay in receiving the needed attention when the health workers were called.

“*I think they should improve upon their care*, *especially the night nurses*. *I don’t know whether they are too less or too many*. *It is as if when they come for the night-duty they have a fixed mind on what they are coming to do already so it is very difficult for them to come to the patients*. *When you need their services too it takes some time before they attend to you*.”

*(27 years and married)*

Other women described a mixed picture where some of the caregivers offered good care whiles others did not. One participant who was a teenager indicated that, in some situations, health workers ignore or neglect labouring women when they call for help

*“For the theatre I was okay*. *But at the labour ward I was not able to do anything*. *I was just screaming and they were telling me I should calm down*. *Some of the midwives are good and some are not good*. *When you call someone to help you the person will never come*. *They will tell you that it‘s okay*. *I will say the care was good*, *somehow*.*”*

*(18 years and single)*

On the other hand, some of the mothers were pleased with the care they received during the childbirth process and expressed their satisfaction. Some of the mothers experienced excellent and supportive care from the some of the midwives. There were instances when some women were warmly received, had friendly communication with the health workers and experienced anxiety-free care.

“*I had a lot of experiences*. *I was received warmly and taken care of*. *They laugh and chat with us*. *There’s no anxiety here*. *It was a good experience”*

*(36 years and married)*

*“The doctors and nurses did very well*. *I was initially afraid when they told me that my life and that of the baby were at risk but they did very well for me”*

*(32 years and married)*

“*At the labour ward*, *there were wonderful midwives*. *They had a lot of patience for me and they were very supportive*.*”*

*(37 years and married)*

*“When the baby was coming*, *they used to converse with me to take my mind off the pain I was going through*.*”*

*(25 years and married)*

Another important point mentioned by the participants was optimal monitoring during the IOL. Although most of the respondents were monitored regularly and optimally during the induction of labour and childbirth, some mentioned that the health workers had to be called before they came to monitor them. The participants expected to experience “one on one” care especially during the induction process. Similarly, there were several instances when the maternity staff could not examine the women regularly as scheduled unless they were called or prompted.

“*They monitored me on time always*. *They were even sitting by me and they will place their hands on my abdomen to monitor the baby”*

*(36 years and married)*

“*They were checking me regularly*. *But if you don’t go there yourself and call them*, *they won’t come*. *Sometimes the doctor gives you a time that he will come and check you but the time will be up and he won’t come and you have to walk there and go and call them”*

*(18 years and single)*

Some of the mothers felt there were significant unprofessionalism with some of the health workers. One woman felt some of the staff are not aware of the specific conditions the women were being treated for and therefore were not adequately prepared to care for those medical conditions. For instance, she described a situation when she had diabetes in pregnancy and had to tell the nurse to check her blood sugar and even with that the nurse never came back to check on her.

“*Whenever the staff come to work*, *they know that I will do vitals and all*. *Sometimes you will be here and you have to tell them what to do*. *You should know the person’s condition and what to do and should not be told what to do*. *Everyone who comes here says they did not bring gloves and have to go and come but they never come back because they have their own things to do*. *I told one person that I have diabetes so she should check my sugar*. *I waited for a long time and she did not show up again*.*”*

*(39 years and married)*

Failed induction occurred in some of the respondents (n = 5) which resulted in significant anxiety. Few women were dissatisfied about the procedure because the procedure failed and they could not achieve vaginal birth they aimed for. This finding partly relates to inadequate pre-procedure counselling on the potential outcomes of induction of labour. One respondent indicated how angry she became after 24 hours of induction and requested for caesarean section.

*“I got angry about the induction because the day after the induction the baby was not out and I went to them to do CS for me*.*”*

*(28 years and married)*

In addition, some of the women commented they were not adequately informed nor consented for some procedures or services rendered to them.

“*I was there when she asked me to turn for the injection and I did*. *So she didn’t do her work well*.*”*

*(18 years and single)*

*“I think it is with the doctors but they did not give me any reasons*.*”*

*(28 years and married)*

There were significant diverging opinions and experiences concerning the quality of care women received during the IOL and delivery. Some of the respondents (n = 4) reported they received good quality care during their admission at the hospital.

“*On all the days*, *everyone who came talked to me nicely*”

*(31 years and married*)

“*When I delivered my baby*, *because at the time the nurse was telling me to push I did not even know what was going on and they encouraged me*. *Before I realised they were asking me to look at the sex and tell whether it is a male or female*.*”*

*(26 years and married)*

One woman mentioned that although the health workers did not do anything extra-ordinary, she appreciated the frequency of monitoring she received. This comment indicates women expect regular monitoring by health workers during childbirth.

“*They did not do anything out of the ordinary but the frequency with which they came to check up on me made me appreciate them*.*”*

*(32 years and married)*

The presence of multiple health workers during childbirth undertaking specific patient monitoring roles and ensuring safety of both the mother and baby was considered extremely reassuring by some of the parturient. In describing the experiences of care, one women cited one moment in labour when she had two doctors who were monitoring her, and felt she experienced good quality care.

*“When I went to the labour ward*, *there were two doctors with me and one was checking the baby’s heartbeat while the other did a scan for me*. *That was the moment I felt I had received good quality care*.*”*

*(25 years and married)*

Suboptimal quality care was an important concern that emerged from the narratives of some participants. Nine respondents (52.9%) remarked the care provided was suboptimal. The respondents had various reasons and negative experiences for which they felt they had not received good quality care during their hospital admission. Some of the negative experiences related to procedures and treatment they received include injections.

“*There was one injection that made me bleed and the doctor was monitoring me because they did not know where the blood was coming from*, *but it was from my buttock*”

*(18 years and single)*

“*I was pricked several times when they were setting a line for me*.*”*

*(26 years and married)*

Women’s expectation of care emerged as a recurring theme in most of the narratives. Some of the respondents narrated mixed experiences during the induction process and childbirth. Two respondents had their expectations care unmet. To one, it was because she was disrespected by a health worker and the other thought the nurses did not render the needed help on time. There were instances that the midwives did not respond to the women in labour when they call for help.

“*Some of the nurse disrespected me so the care I expected to receive*, *I did not get*.*”*

*(18 years and single)*

*“I was not expecting anything big just the normal thing like showing concerns to your patients*, *attending to the patients*, *giving them the right medications*. *I don’t know if when we shout they don’t hear us*. *She will have to go and call them and come because you will shout ‘nurse’ and no one will come*. *But when you go out there you get someone to come*.*”*

*(27 years and married)*

For the remaining patients, the care expectations were met and even exceeded for some. Some of the participants confirmed that the care they received from the health workers were consistent with their expectations. Originally, some were not expecting to receive high level of care but they received good quality of care from the health workers.

“*I got what I expected*. *They spoke to me well and that even helped my BP that had been going up*.*”*

*(32 years and married)*

“*I received the care but the induction failed*. *I had heard some things about nurses here that they were stern and impolite but that was not what I experienced*.*”*

*(28 years and married)*

### 3. Women’s difficult experiences and coping strategies

Some of the women indicated the vaginal examination process was the most difficult or painful experience they encountered during the process of labour induction and childbirth. Most participants devised strategies to endure the painful experience such as psyching themselves for the vaginal examination as they had no other choice. Vaginal examination including insertion of the medication vaginally for IOL was described as uncomfortable and extremely painful.

“*It was the pains I was experiencing from the inserted medicine*, *and the operation*. *But the medicine was more painful*.*”*

*(32 years and married)*

“*It is the pain*. *I was uncomfortable when the doctor inserted the medication but I did not have a choice*. *The most difficult part was the pain*”

*(35 years and married)*

“*The point where they were inserting the hands in my private part me was painful*”

*(31 years and married)*

In addition, most women described the labour pain following induction of labour as extremely excruciating. One woman who had experienced spontaneous labour in her previous childbirth compared the painful experience of labour induction with spontaneous labour and described the former as more painful. In addition, the process of inserting the medication intravaginally was considered distasteful and traumatic and were performed frequently.

“*The induction is painful*, *compared to normal labour*. *The insertion of the medicine is painful and it was frequent*”

*(25 years and married)*

However, few women did not find the process of induction of labour painful because they had psyched themselves for the whole procedure and were ready for any associated challenges. One woman indicated that the whole induction process was for the safety of her baby and herself and therefore did not find anything challenging about the procedure. These comments buttress the psychological and physical trauma associated with vaginal examination during induction of labour and childbirth for which most women had to prepare themselves to endure.

“*I did not find any aspect to be difficult because I psyched myself up*.*”*

*(39 years and married)*

“*I knew it was all for my good and my baby’s good so I did not find anything difficult*.*”*

*(36 years and married)*

One woman stated that she did not find any part of the process challenging; however, she quickly added she experienced some pains during the vaginal assessment but she conditioned her mind to go through the examination. It is evident that an important frequently emerged sub-theme was the coping strategies devised by the women during vaginal examination for the IOL. Most women psyched themselves for the dreaded vaginal examination associated with the IOL and childbirth

“*There was no difficult part in the care*. *I felt some pains during the insertion of the drug but I had tuned my mind to go through it*.*”*

*(36 years and married)*

## Discussion

In this qualitative study, women’s lived experiences of care during labour induction and childbirth were explored. three broad themes emerged from the narratives: women’s knowledge about induction of labour, women’s experiences with care, and women’s difficult experiences and coping mechanisms. In general, we determined mixed responses from the respondents in all the themes explored: adequate versus inadequate knowledge; positive and negative experiences of care; good and suboptimal quality of care; challenges with vaginal examinations and coping strategies.

We found that nearly half of the women had no prior knowledge about induction of labour and only heard of the intervention at the time the procedure was undertaken. Close to 60% had some knowledge about IOL prior to undergoing the procedure with varied sources of information: health workers, other people and from the internet. In recent times, the internet has become the commonest source of health information for most people [28, 29]. For instance, most pregnant women obtain information about their birth plans and other health education from the internet especially in the high-income settings [29]. The knowledge gap determined in this study is partly attributed to the low rate of induction of labour, inadequate maternal health education, low literacy and low patronage of internet by the general women population in our subregion [30]. In this study, only one woman had previously experienced induction of labour and therefore had adequate knowledge about it. Nearly all respondents, however, reported that they were counselled before the procedure was commenced. In a study involving two groups of women who were being prepared for induction of labour, one group was counselled using a standard counselling tool and the other was counselled using a non-standard tool. The study indicated the first group had a better knowledge and understanding about the procedure, even though outcome of the procedure had no direct relation to their understanding [31].

Inadequate knowledge about induction of labour may impact negatively on women’s experiences and satisfaction with care. In this study, a mixed picture of satisfaction and dissatisfaction was reported by the women suggestive of considerable burden of sub-optimal care. Some of the women who underwent induction of labour expressed their dissatisfaction with the care, partly attributed to inadequate pre-procedure counselling. Reasons for dissatisfaction cited by the women related to neglect by the midwives especially during night shifts where some mothers felt they were ignored when they needed urgent attention. In addition, adequate pre-procedure education, counselling and shared decision making including informed consent are viable measures to improve women’s confidence and satisfaction. For instance, a recent WHO multi-country study on mistreatment determined that nearly 27% of women who underwent IOL did not provided informed consent [11]. Other reasons included the roster schemes of midwives/nurses that assign fewer numbers to night duties compared to morning and afternoon duties or actual lack of adequate number of health workers might have contributed to women’s experiences of dissatisfaction with the care they received. Similarly, Sandovski et al reported that women are generally satisfied with the care received from morning staff compared with afternoon and night staff [32] as explored in our study. In another study in Ghana, it was found that clients’ satisfaction in maternal care was linked to how thorough they were examined, and how they were treated with courtesy and respect [33]. Also, another study in Ghana determined that patients expressed their dissatisfaction at the poor attitudes towards work and poor conditions of some logistics [34]. In this study, majority of the respondents narrated how they were monitored regularly during the induction and childbirth processes; however, there were instances where women reported their experiences of substandard care.

Similarly, there were divergent views concerning the quality of care received at the health facility. The focus of opinions related to the frequency at which they were reviewed by doctors, the number of doctors and midwives that attended to them. The individual experiences varied because the facility has different workers with different levels of competencies against which the participants weighed their expectations of care. Majority of the respondents however were satisfied with the care they received. One respondent was disappointed because she had failed induction of labour and demanded for caesarean section but her request was not honored by the health workers. Failed induction is the commonest complication of induction of labour and is associated with 2–3 times increase in caesarean section rates [35–37]. In this study failed IOL occurred in five pregnant women who were finally delivered via caesarean section. In current era of increasing caesarean section rates, careful client selection and adequate feto-maternal monitoring during the process of labour induction can potentially minimize caesarean section rates and simultaneously optimize labour outcomes.

Generally, vaginal examination is recommended as an important clinical assessment for monitoring the progress of labour to decide the timing of obstetric interventions. However, there is evidence of significant negative experiences reported by women such as severe pain, discomfort, embarrassment and fear during vaginal examinations [23, 24, 38]. In this study, some of the women mentioned that their most difficult experience during the induction process was vaginal examination, mainly because it was painful and uncomfortable. Few women who found the procedure less bothersome remarked that they psyched themselves for the vaginal examination but it was painful. Generally, the initial vaginal examination for induction procedure may involve cervical assessment and or administration of the pharmacological agent, most likely vaginal misoprostol. However, oral misoprostol has been found to have comparable safety and efficacy for induction and is recommended by the World Health Organization [2]. More recent multi-country evidence indicates that women experience varied types of mistreatments during vaginal examination at health facilities during childbirth [11, 22]. Moving forward, the use of oral misoprostol is recommended as a viable alternative to minimize the pain and severe psychological trauma experienced by women during vaginal examination for women undergoing induction of labour.

### Strengths and limitations

The study’s greatest strength relates to its qualitative methodology using in-depth interviews, which enabled the women to offer their firsthand accounts of the care they received, emphasizing the difficulties and important suggestions for care improvement. The use of in-depth interviews provided the mother the opportunity to describe the details of their personal experiences. The interviews were done following the women’s hospital discharge (exit interviews). The exit interviews provided the respondents an unrestricted opportunity to discuss their lived experiences without worrying that they might receive subpar care. In addition, we included women with varied obstetric characteristics (nulliparous versus multiparous) and managed by different groups of maternal health specialists resulting in a comprehensive case mix.

The study’s shortcomings include the use of a single tertiary hospital as opposed to a multicenter design, which would have provided a more comprehensive picture of women’s experiences of induction of labour in the country. The study site is the largest tertiary center in Ghana where the quality of care is expected to be highest. The experiences of women in secondary or other lower cadre facilities and private hospital may be significantly different from the tertiary center where the study was conducted. Also, the experiences of women who had spontaneous onset of labor were not included in this qualitative synthesis. The exclusion of spontaneously achieved labor counterparts potentially limits the comparability of the extent of care experiences of women who underwent labor induction.

Additionally, using a single interviewer is considered a limitation compared with the use of two or more interviewers with varied expertise. The interviewer had the requisite training, skills and experience in qualitative data collection to optimize the data collection.

## Conclusion

Our study has highlighted women’s lived experiences of care during labour induction and childbirth at a tertiary health facility in Ghana. There were mixed findings concerning women’s knowledge about induction of labour (adequate versus inadequate knowledge), their satisfaction with care and quality of care received. Women’s most difficult experiences related to frequent vaginal examination which were variously described: traumatic, painful and uncomfortable. Nearly all women employed various coping mechanisms and strategies including psyching themselves and self-motivation to navigate and endure various forms of negative and painful experiences during induction of labour and childbirth.

To improve women’s experiences, expectations and satisfaction with care, appropriate antenatal counselling, women empowerment and pre-labour education on childbirth processes and expectations are recommended to enhance their birth preparedness and complication awareness. Similarly, regular and tailored refreshers courses and continuous professional development are recommended for health workers to better understand the perspectives and experiences of laboring women. Health system re-structuring with special focus on women-centered care and shared decision is strongly recommended in the facility and similar settings.

Further research is recommended but in a multicentered setting, with appropriate implementation of a standardized counselling guide for the prospective induction of labour clients to facilitate positive childbirth experiences.

## Supporting information

S1 Text Guide for in-depth interview. (DOCX)
