# Supplementary material for: Lived experiences of women during induction of labour at a tertiary hospital in Ghana: A qualitative study
Source: PLOS Glob Public Health. 2024 Feb 15;4(2):e0002290. doi: 10.1371/journal.pgph.0002290 (PMC10868737; doi:10.1371/journal.pgph.0002290)
Supplement: S1 Text — (DOCX) [file pgph.0002290.s001.docx]

**Interview guide for women who underwent induction of labour**

1. Could you describe why you were admitted to the hospital?
2. Could you describe how you were cared for during your admission at the hospital?
3. How did you experience the care you received at the hospital?
4. How did your admission at the hospital affect you and your family?
5. During your admission you’ve interacted with others (Probe further: medical professionals, your family, other patients) how do you relate to them? Did any conflicts occur, why?
6. What is the most difficult aspect of the care you received at the hospital? (probe further)
7. In your opinion, did you experience any disrespectful care (Probe further: verbal abuse, physical abuse, discrimination, neglect, professionalism etc)?
8. Prior to the procedure, did you know about induction of labour? (probe further)
9. Do you think there were adequate number of doctors, nurses and other professionals available to take care of you and other patients? (probe further)
10. In your opinion, did you receive the care you expected to receive at the hospital? (probe further)
11. In future, would you prefer to be cared for again in the hospital or you would prefer elsewhere if you had the opportunity? (probe further)
12. Do you think you were checked or monitored regularly as expected during the process of induction? (probe further)
13. Do you think you were able to do all the laboratory tests requested for you? (probe further)
14. What recommendations do you suggest to help improve the quality of care for women undergoing induction of labour at the hospital?
